# Dental and periodontal disease in patients with inflammatory bowel disease

**DOI:** 10.1007/s00784-021-03835-6

**Published:** 2021-02-23

**Authors:** Christopher X. W. Tan, Henk S. Brand, Bilgin Kalender, Nanne K. H. De Boer, Tymour Forouzanfar, Jan G. A. M. de Visscher

**Affiliations:** 1 Department of Oral and Maxillofacial Surgery/Oral Pathology, Amsterdam University Medical Centre/Academic Centre for Dentistry Amsterdam (ACTA), PO Box 7057, 1007 MB Amsterdam, The Netherlands; 2 Department of Oral Biochemistry, Academic Centre for Dentistry Amsterdam (ACTA), Amsterdam, The Netherlands; 3 Department of Gastroenterology and Hepatology, Amsterdam University Medical Centre, VU University, AGEM Research Institute, Amsterdam, The Netherlands

**Keywords:** Inflammatory bowel disease, Oral health, DPSI (Dutch Periodontal Screening Index), DMFT (Decayed, Missing, Filled Teeth), Crohn’s disease, Ulcerative colitis

## Abstract

**Objectives:**

Although bowel symptoms are often predominant, inflammatory bowel disease (IBD) patients can have several oral manifestations. The aim of this study was to investigate the prevalence of dental caries and periodontal disease in patients with Crohn’s disease (CD) and ulcerative colitis (UC) compared to an age and gender-matched control group of patients without IBD.

**Material and methods:**

The DMFT (Decayed, Missing, Filled Teeth) scores and the DPSI (Dutch Periodontal Screening Index) of 229 IBD patients were retrieved from the electronic health record patient database axiUm at the Academic Centre for Dentistry Amsterdam (ACTA) and were compared to the DMFT scores and DPSI from age and gender-matched non-IBD patients from the same database.

**Results:**

The total DMFT index was significantly higher in the IBD group compared to the control group. When CD and UC were analyzed separately, a statistically significant increased DMFT index was observed in CD patients but not in UC patients. The DPSI did not differ significantly between the IBD and non-IBD groups for each of the sextants. However, in every sextant, IBD patients were more frequently edentulous compared to the control patients.

**Conclusion:**

CD patients have significantly more dental health problems compared to a control group. Periodontal disease did not differ significantly between IBD and non-IBD groups as determined by the DPSI.

**Clinical relevance:**

It is important that IBD patients and physicians are instructed about the correlation between their disease and oral health problems. Strict oral hygiene and preventive dental care such as more frequent checkups should be emphasized by dental clinicians.

## Introduction

Inflammatory bowel diseases (IBD) are chronic, immune-mediated diseases of the gastrointestinal tract [1]. The exact pathogenesis is unknown, but an interaction of host susceptibility and environmental triggers appears likely [2]. The two major types of IBD are Crohn’s disease (CD) and ulcerative colitis (UC). CD can affect any part of the gastrointestinal tract, while UC primarily affects the rectum and may extend proximally up to the entire colon [3]. The overall worldwide incidence of CD and UC ranges depending on the region from 0.0 to 29.3 and 0.15 to 57.9 per 100.000 person-years respectively [4]. Bowel symptoms are predominant, but also a variety of extra-intestinal manifestations including those affecting the oral cavity can appear. Oral manifestations reported in IBD patients are aphthous ulcerations, cobblestoning of the oral mucosa, orofacial swelling, and pyostomatitis vegetans (mainly in UC), but IBD patients also appear to have an increased risk for dental caries, periodontitis, and xerostomia [5–8]. These manifestations may coincide, precede, or follow at any time during the intestinal symptoms [9–11].

Little is known about the dental and periodontal status of patients with IBD. The aim of this study was to investigate the prevalence of dental caries and periodontal disease in patients with CD and UC compared to an age and gender-matched control group of patients without IBD.

## Material and methods

### Patient selection

In this retrospective study, data were retrieved from the electronic health record patient database axiUm (Exan group, Coquitlam, British Columbia, Canada) at the Academic Centre for Dentistry Amsterdam (ACTA). This database contains individual records of patients registered at ACTA since January 1, 1998. All patients visiting ACTA are obligated to fill in questionnaires including their medical history according to the European Medical Risk-Related History (EMRRH) questionnaire [12]. These data were used to retrieve patients having CD or UC. For every patient with either CD or UC, a non-IBD patient with the same age and gender was randomly selected from the electronic health record database. This non-IBD patient was matched with regard to age and gender, differed maximally 1 month of age from the IBD patient, and visited ACTA in the same year as the matched IBD patient.

### Data extraction

#### Dental assessment

The DMFT index (Decayed, Missing, Filled Teeth) was extracted from the dental charts according to the criteria of the World Health Organization [13]. The DMFT index is the sum of the number of decayed (D), missing (M), and filled (F) teeth (T). Missing and filled teeth as a result of trauma were not included. In addition to the total DMFT index, the DMFT index was registered for 6 subregions: upper front region, upper premolar region, upper molar region, lower front region, lower premolar region, and lower molar region. Edentulous IBD patients were excluded from the dental assessment.

#### Periodontal assessment

The DPSI (Dutch Periodontal Screening Index) was retrieved from the most recent checkup note or from the last periodontal chart and was registered for each sextant [14]. The DPSI is a scoring system where the severity of periodontitis is scored for each sextant on a scale from 0, 1, 2, 3−, 3+, 4, and *X* (see Table 8). Edentulous IBD patients were excluded from the periodontal assessment.

If the DMFT index or DPSI of a patient with IBD was not retrievable, this information was also left out for the matching control subject. Several potential covariates were extracted from the medical assessment form: age, gender, diabetes mellitus, xerostomia, smoking, daily intake of alcohol, and the use of IBD-related medication (corticosteroids, biologicals, immunosuppressants, and aminosalicylates). Details on medication are given in Table 9.

### Statistical analysis

Data are presented as means ± SD or as percentages and were statistically analyzed using SPSS Statistics for Windows, V.23.0 (Armonk, New York, USA). Data between the groups were compared using the Pearson Chi-square test. For the comparison of DMFT indices between groups, the Mann Whitney *U* test was used. The significance level was set at 0.05.

### Study population

A total of 229 patients with IBD were identified in axiUm and consisted of 133 females (58%) and 96 males (42%), with a mean age of 51 ±16 years (Table 1). In the IBD group, 148 (65%) had CD, 80 (35%) had UC, and 1 (0.5%) reported to have IBD undetermined. The DMFT index of 17 patients was not retrievable (CD *n* = 13, UC *n* = 4), and in 18 cases, the DPSI was unknown (CD *n* = 12, UC *n* = 6). There were no significant differences in the prevalence of diabetes mellitus between the IBD and non-IBD groups; in both groups, the percentage of patients with diabetes mellitus was 6.6%. Subjects in the non-IBD group smoked significantly more frequently than subjects in the IBD group. There was no significant difference in the daily intake of alcohol between IBD patients and controls. More than half of the IBD patients used IBD-related medication while the control subjects seldom used these medications. A total of 36 IBD patients used corticosteroids, 27 biologicals, 25 immunosuppressants, and 59 aminosalicylates. In the control group, 1 patient used an immunosuppressant, 2 corticosteroids, and 1 patient used a biological (Table 1).

**Table 1:** Demographic characteristics of IBD patients (*n* = 229) and non-IBD controls (*n* = 229)

	IBD	Non-IBD	*p*-value
Male	96	96	
Female	133	133	
Mean age (years)	51 ± 16	51 ± 16	
Crohn’s disease			
Male	60		
Female	88		
Total	148		
Ulcerative colitis			
Male	36		
Female	44		
Total	80		
IBD undetermined	1		
Diabetes mellitus	15	15	
Smoking	53	72	0.046
Daily intake of alcohol	19	17	0.728
Use of IBD-related medication	125	4	<0.0005

## Results

### Dental assessment

The total DMFT index was significantly higher in the IBD group compared to the control group (Table 2). When stratified for 4 dental subregions in the upper and lower jaw, this difference remained significant for 3 subregions except for the upper premolar and molar region. In CD patients, the total DMFT index and the DMFT scores of the upper front, lower front, and lower premolar and molar regions were significantly higher than in the control group (Table 3). For the upper premolar and molar region, this difference almost reached statistical significance (*p* = 0.076). There were no significant differences in total DMFT index and in DMFT scores for the 4 dental subregions in UC patients compared to the control group (Table 4). There were no significant differences in DMFT scores in patients with or without the use of IBD-related medication.

**Table 2:** Total DMFT indices of IBD patients (*n* = 212) and controls (*n* = 212), stratified for all and 4 oral regions. DMFT data are presented as mean ± SD

	IBD	Non-IBD	*p*-value
DMFT total	14.3 ± 7.8	12.3 ± 7.0	0.012
DMFT upper front region	2.5 ± 2.4	1.9 ± 2.3	0.013
DMFT upper (pre)molar region	5.6 ± 2.6	5.3 ± 2.6	0.135
DMFT lower front region	1.0 ± 1.9	0.5 ± 1.3	<0.0005
DMFT lower (pre)molar region	5.2 ± 2.4	4.7 ± 2.5	0.023

**Table 3:** DMFT indices of CD patients (*n* = 135) and controls (*n* = 135), stratified for all and 4 oral regions. DMFT data are presented as mean ± SD

DMFT	CD	Non-IBD	*p*-value
DMFT total	14.6 ± 8.0	11.8 ± 7.1	0.002
DMFT upper front region	2.6 ± 2.5	1.8 ± 2.2	0.004
DMFT upper (pre)molar region	5.7 ± 2.7	5.1 ± 2.7	0.076
DMFT lower front region	1.1 ± 1.9	0.4 ± 1.3	0.001
DMFT lower (pre)molar region	5.3 ± 2.5	4.5 ± 2.5	0.016

**Table 4:** DMFT indices of UC patients (*n* = 76) and controls (*n* = 76), stratified for all and 4 oral regions. DMFT data are presented as mean ± SD

DMFT	UC	Non-IBD	*p*-value
DMFT total	13.8 ± 7.5	13.2 ± 6.8	0.643
DMFT upper front region	2.2 ± 2.4	2.0 ± 2.3	0.628
DMFT upper (pre)molar region	5.5 ± 2.6	5.6 ± 2.4	0.748
DMFT lower front region	0.9 ± 1.8	0.6 ± 1.4	0.287
DMFT lower (pre)molar region	5.2 ± 2.4	4.9 ± 2.4	0.591

### Periodontal assessment

The DPSI did not differ significantly between the IBD and non-IBD groups for each of the sextants (Table 5). However, in every sextant, IBD patients were more frequently edentulous compared to the control patients. There was no significant difference in DPSI for both CD and UC patients when compared to their respective control patients (Tables 6 and 7).

**Table 5:** DPSI scores of IBD patients (*n* = 211) compared to the control group (*n* = 211). DPSI scores 0, 1, and 2 were combined because these categories indicate no clinical attachment loss. The DPSI scores and the percentage of edentulism in each sextant were compared using Chi-square tests

DPSI	≤ 2	3−	3+	4	*p*-value	Edentulous	*p*-value
1st sextant control	83 (39%)	61 (29%)	17 (8%)	34 (16%)	0.539	16 (8%)	0.040
1st sextant IBD	78 (37%)	50 (24%)	11 (5%)	43 (20%)		29 (14%)	
2nd sextant control	137 (65%)	39 (19%)	15 (7%)	11 (5%)	0.409	9 (4%)	0.003
2nd sextant IBD	129 (61%)	32 (15%)	8 (4%)	16 (8%)		26 (12%)	
3rd sextant control	88 (42%)	59 (28%)	18 (9%)	32 (15%)	0.955	14 (7%)	0.016
3rd sextant IBD	79 (37%)	61 (29%)	16 (8%)	26 (12%)		29 (14%)	
4th sextant control	100 (47%)	60 (28%)	15 (7%)	31 (15%)	0.664	5 (2%)	0.001
4th sextant IBD	82 (39%)	64 (30%)	19 (9%)	24 (11%)		22 (10%)	
5th sextant control	166 (79%)	18 (9%)	12 (6%)	12 (6%)	0.087	3 (1%)	0.018
5th sextant IBD	155 (74%)	25 (12%)	5 (2%)	14 (7%)		12 (6%)	
6th sextant control	103 (48%)	55 (26%)	13 (6%)	35 (17%)	0.574	5 (2%)	0.005
6th sextant IBD	90 (43%)	62 (29%)	14 (7%)	27 (13%)		18 (9%)

**Table 6:** DPSI scores of CD patients (*n* = 136) compared to the control group (*n* = 136). DPSI scores 0, 1, and 2 were combined because these categories indicate no clinical attachment loss. The DPSI scores and the percentage of edentulism in each sextant was compared using Chi-square tests

DPSI	≤ 2	3−	3+	4	*p*-value	Edentulous	*p*-value
1st sextant control	57 (42%)	43 (32%)	9 (7%)	19 (14%)	0.903	8 (6%)	0.017
1st sextant CD	48 (35%)	36 (27%)	8 (6%)	24 (18%)		20 (15%)	
2nd sextant control	90 (66%)	27 (20%)	8 (6%)	6 (4%)	0.578	5 (4%)	0.008
2nd sextant CD	80 (59%)	22 (16%)	7 (5%)	10 (7%)		17 (13%)	
3rd sextant control	59 (43%)	42 (31%)	10 (7%)	18 (13%)	0.754	7 (5%)	0.013
3rd sextant CD	48 (35%)	41 (30%)	11 (8%)	17 (13%)		19 (14%)	
4th sextant control	65 (48%)	43 (32%)	6 (4%)	19 (14%)	0.125	3 (2%)	0.003
4th sextant CD	52 (38%)	43 (32%)	15 (11%)	11 (8%)		15 (11%)	
5th sextant control	110 (81%)	13 (10%)	5 (4%)	6 (4%)	0.522	2 (2%)	0.053
5th sextant CD	101 (74%)	15 (11%)	4 (3%)	8 (6%)		8 (6%)	
6th sextant control	63 (46%)	42 (31%)	11 (8%)	17 (13%)	0.817	3 (2%)	0.028
6th sextant CD	58 (43%)	44 (32%)	10 (7%)	13 (10%)		11 (8%)

**Table 7:** DPSI scores of UC patients (*n* = 74) compared to the control group (*n* = 74). DPSI scores 0, 1, and 2 were combined because these categories indicate no clinical attachment loss. The DPSI scores and the percentage of edentulism in each sextant was compared using Chi-square tests

DPSI	≤ 2	3−	3+	4	*p*-value	Edentulous	*p*-value
1st sextant control	25 (34%)	18 (24%)	8 (11%)	15 (20%)	0.245	8 (11%)	0.797
1st sextant UC	30 (41%)	14 (19%)	2 (3%)	19 (26%)		9 (12%)	
2nd sextant control	46 (62%)	12 (16%)	7 (10%)	5 (7%)	0.398	4 (5%)	0.147
2nd sextant UC	48 (65%)	10 (14%)	1 (1%)	6 (8%)		9 (12%)	
3rd sextant control	29 (39%)	16 (22%)	8 (11%)	14 (19%)	0.659	7 (10%)	0.439
3rd sextant UC	30 (41%)	20 (27%)	5 (7%)	9 (12%)		10 (14%)	
4th sextant control	35 (47%)	16 (22%)	9 (12%)	12 (16%)	0.492	2 (3%)	0.085
4th sextant UC	29 (39%)	21 (28%)	4 (5%)	13 (18%)		7 (10%)	
5th sextant control	55 (74%)	5 (7%)	7 (10%)	6 (8%)	0.149	1 (1%)	0.172
5th sextant UC	53 (72%)	10 (14%)	1 (1%)	6 (8%)		4 (5%)	
6th sextant control	39 (53%)	13 (18%)	2 (3%)	18 (24%)	0.511	2 (3%)	0.085
6th sextant UC	31 (42%)	18 (24%)	4 (5%)	14 (19%)		7 (10%)	

The incidence of edentulism in CD patients was significantly higher compared to controls in the 1st, 2nd, 3rd, 4th, and 6th sextant (Table 6). UC patients did not demonstrate a significantly higher incidence of edentulism for any sextant when compared to control patients (Table 7). There were no significant differences in DPSI and edentulism in patients with or without the use of IBD-related medication.

Xerostomia was significantly more frequently reported in IBD patients compared to the control group (8.8% vs. 2.6%, *p* = 0.005). This difference was significant for CD (8.8% vs. 2.0%, *p* = 0.010), but not for UC (8.6% vs. 3.7%, *p* = 0.192). Xerostomia was not significantly related with the use of IBD-related medication (*p* = 0.815).

## Discussion

This study investigated dental and periodontal disease in patients with IBD. Dentate CD patients had a higher DMFT index than the control group. There were no significant differences in the DMFT index of UC patients compared to the control group. There were no significant differences in periodontal disease as determined by the DPSI between dentate IBD patients and the control group. Both CD and UC patients did not show any significant differences in DPSI when compared to the control group.

As a small dental restoration equally contributes to the DMFT index as a missing tooth, this could mean that IBD patients potentially still have the same number of functional teeth as control subjects. However, analyzing the DPSI data, it was shown that IBD patients had significantly more edentulous sextants than control subjects (Table 5). This is in contrast with a reported study that found no significant difference in the number of teeth present between IBD and non-IBD patients [8]. A possible explanation may be that their patient group was considerably younger than the IBD population in the present study (mean age 38.4 ± 10.2 vs. 51 ± 16 years), which may have contributed to the differences in missing teeth. An additional argument for that is that the same study found a higher prevalence of dentine caries in IBD patients (40% vs. 22% in the controls) which might be a reason for tooth loss with aging. A more recent study investigated a cohort more similar in age to the present study (mean age; CD 53.1 ± 10.3 years, UC 57.0 ± 8.2 years) and reported that IBD patients needed more treatments to prevent tooth loss [15]. This is in accordance with findings in the present study.

Several studies have reported an increased DMFT index for CD patients [16–19]. It has been suggested that patients with CD may have a higher incidence of dental caries because of nutritional deficiencies, changes in salivary conditions, and oral microflora [20]. CD patients have increased numbers of *Lactobacilli* and *Streptococcus mutans* in the oral cavity which seems to be related to a more frequent intake of refined sugars by CD patients [8, 16, 20–22]. In the present study, xerostomia was significantly more frequently reported by CD patients compared to the controls. Although xerostomia is not always related to hyposalivation, previous studies have shown that CD and UC patients can have hyposalivation in resting and chewing-stimulated conditions and that the composition of the saliva in CD patients may be correlated to bowel disease activity [7, 23]. A decreased salivary flow increases the risk of developing dental caries and might have contributed to the increased DMFT index in patients with CD [24].

Only three previous studies reported a significantly higher DMFT index in UC patients [17–19]. These studies were performed in China, Brazil, and Greece, so dissimilarities in comparison to a western European population cannot be excluded. Furthermore, in the Greek study, only children and adolescents were investigated while in the present study, only adult patients were included [18]. The Brazilian study adjusted in the statistical analysis for plaque levels [17]. In the Greek study, no significant differences in plaque scores were found. As plaque scores were not included in the present study, we cannot exclude the possibility that the control subjects may had higher plaque scores than UC patients. Another study reports higher caries treatment needs in UC patients when compared with controls, but the differences were less clear than in patients with CD and it was speculated that this was caused by a higher sugar intake in CD patients compared to UC patients [15, 21, 25].

Periodontitis and IBD are both considered to be a disproportionate mucosal inflammatory response to micro-organisms in susceptible patients. Recent reviews of epidemiological studies conclude that there seems to be increasing evidence for a correlation between IBD and periodontal disease [19, 26–28]. The present study failed to produce evidence for a correlation between IBD and periodontal disease. When CD and UC groups were compared separately with the controls, there was no significant difference in the DPSI between the patients and their controls. Also, the clinically higher scores 3+ and 4, which indicate clinical attachment loss and thus indicate periodontitis, were not significantly different. A higher prevalence of periodontitis was reported in IBD patients; however, smoking turned out to be an effective modifier since there was no difference in the prevalence of periodontitis among non-smoking control patients and non-smoking patients with IBD [17]. It was found that clinical signs of gingivitis and periodontitis were higher among IBD patients, but that not smoking decreased the risk of periodontitis [29]. In the present study, control patients smoked significantly more than IBD patients which might explain why the DPSI is not significantly different. As smoking is a known risk factor for periodontitis, we have to interpret the findings of this study regarding the DPSI with caution [30, 31]. An increased prevalence and severity of periodontal disease for IBD patients was reported; however, this study was performed in a Middle-Eastern population with a poor average level of oral hygiene, as more than 20% of the included IBD patients stated that they never had brushed their teeth [32]. Other studies found results comparable to the present study and also showed no significant differences in periodontal disease between IBD and non-IBD groups [8, 15]. It should be taken into consideration that various other factors such as oral hygiene, poorly controlled diabetes, and smoking history are also risk factors for periodontal disease [33, 34] and these factors differed considerable between the previously discussed studies. Furthermore, different methods were used for the assessment of the absence, and presence, and the degree of periodontal disease. To our knowledge, the present study is the first that used DPSI scores in IBD patients. The DPSI is a relatively easy and fast screening method to determine periodontal disease which makes it ideal for routine dental checkups but due to its low specificity, it is not well suited for epidemiological studies [14]. This is because the site with the highest probing depth determines the score for the whole sextant. A patient who has only one site with a periodontal pocket depth of e.g. 6 mm per sextant therefore has the same score as someone who has multiple sites per sextant with the same pocket depth. A full periodontal status would give much more detailed information about the actual severity of periodontal disease.

The present study has several limitations. One of the most important is the retrospective design. The data extracted from the patient records had not been gathered specifically for this study. The dental records were not complete for all patients with IBD and in many cases did not contain information about the current oral hygiene status, plaque index, and dietary habits of the patient. Although registered whether a patient was smoking or not, the number of pack-years was not registered. Oral hygiene has a crucial impact on the DMFT index and the DPSI. For instance, the use of interdental cleaning aids has a huge role in preventing dental decay and periodontal diseases. Some studies have suggested a higher plaque index and bad dietary habits in IBD patients [8, 16, 32]. A higher plaque index in IBD patients was attributed to insufficient oral hygiene because of painful oral manifestations [8]. Therefore, future studies on the influence of oral hygiene habits on dental and periodontal diseases of IBD patients seem warranted.

Statistical significance depends on sample size and expected effect. Recent studies reported statistically significant differences for DMFS and periodontal disease in patients with UC compared to healthy controls [19, 28]. The fact that no significant differences were observed for patients with UC in the present study might be related to the relatively small size of this subgroup of IBD patients. Therefore, a multi-center trial to explore DMFS and periodontal disease in patients with UC seems warranted.

Another possible limitation is that the presented periodontal data are based on the most recent dental evaluation rather than the initial periodontal evaluation during the initial visit of the patients. It could be possible that patients with periodontal disease already had received periodontal therapy and were under supportive therapy during the periodontal evaluation on the last dental checkup. However, as this also applies to non-IBD group, both groups are comparable in this aspect. Nevertheless, it will be interesting to investigate the initial periodontal evaluation with full-mouth measurements of pocket depth, clinical attachment loss, bleeding on probing, and plaque index in future clinical studies.

ACTA is an academic dental school, where most dental checkups are performed by a large number of students. As formal clinical calibration between these students is lacking, this could also have introduced for some inconsistencies in the data.

Despite the limitations, the present study did show that CD patients had a significantly higher DMFT index compared to a control group. IBD exhibits how a systemic disease can complicate and provoke predisposing factors. Hence, it is important that these patients are instructed about the correlation between their disease and dental health problems. Strict oral hygiene and preventive dental care such as more frequent checkups should be emphasized by dental clinicians.

## Appendix 1

**Table 8:** The Dutch Periodontal Screening Index scoring system

DPSI	
0	- Probing depth ≤ 3mm - No bleeding on probing - No dental tartar - No overhanging restorations
1	Same as 0, but bleeding on probing
2	Same as 1, but with dental tartar and/or overhanging restorations
3−	Probing depth of 4-5 mm without gingival recession
3+	Probing depth of 4-5 mm with gingival recession
4	Probing depth ≥ 6mm
*X*	Edentulous

1st sextant: right upper (pre)molar region, 2nd sextant: upper front region, 3rd sextant: left upper (pre)molar region, 4th sextant: left lower (pre)molar region, 5th sextant: lower front region, 6th sextant: right lower (pre)molar region

## Appendix 2

**Table 9:** List of medication used in treatment of inflammatory bowel disease in the Netherlands

Corticosteroids	Biologicals	Immunosuppressants	Aminosalicylates
Beclometasone	Adalimumab	Azathioprine	Mesalazine
Betamethasone	Golimumab	Mercaptopurine	Olsalazine
Budesonide	Infliximab	Tioguanine	Sulfasalazine
Dexamethasone	Vedolizumab	Methotrexate	Salazopyrine
Methylprednisolone	Ustekinumab	Tacrolimus	
Prednisolone		Ciclosporine	
Prednisone			
Triamcinolone			
Triamcinolonacetonide			
Hydrocortisone			
Cortiment			
Entocort			
